# Supplementary material for: Qualitative Analysis Related to Palynological Characterization and Biological Evaluation of Propolis from Prespa National Park (Greece)
Source: Molecules. 2022 Oct 18;27(20):7018. doi: 10.3390/molecules27207018 (PMC9609274; doi:10.3390/molecules27207018)
Supplement: Supplementary file 1 [file molecules-27-07018-s001.zip › molecules-1963763-supplementary.pdf]

Qualitative Analysis Related to Palynological Characterization and Biological Evaluation of Propolis from Prespa National Park (Greece)

Elisavet Pyrgioti [1], Konstantia Graikou [1], Nektarios Aligiannis [1], Karabournioti Sofia [2], Ioanna Chinou [1,*]

[1] Lab of Pharmacognosy and Chemistry of Natural Products, Faculty of Pharmacy National and Kapodistrian University of Athens, Greece. ellipirgioti@gmail.com; kgraikou@pharm.uoa.gr; aligiannis@pharm.uoa.gr

[2] Attiki Bee Culturing Co., Athens, Greece; Kryoneri Attikis, Athens, 14568, Greece. skar@attiki-pittas.gr

* Correspondence: ichinou@pharm.uoa.gr

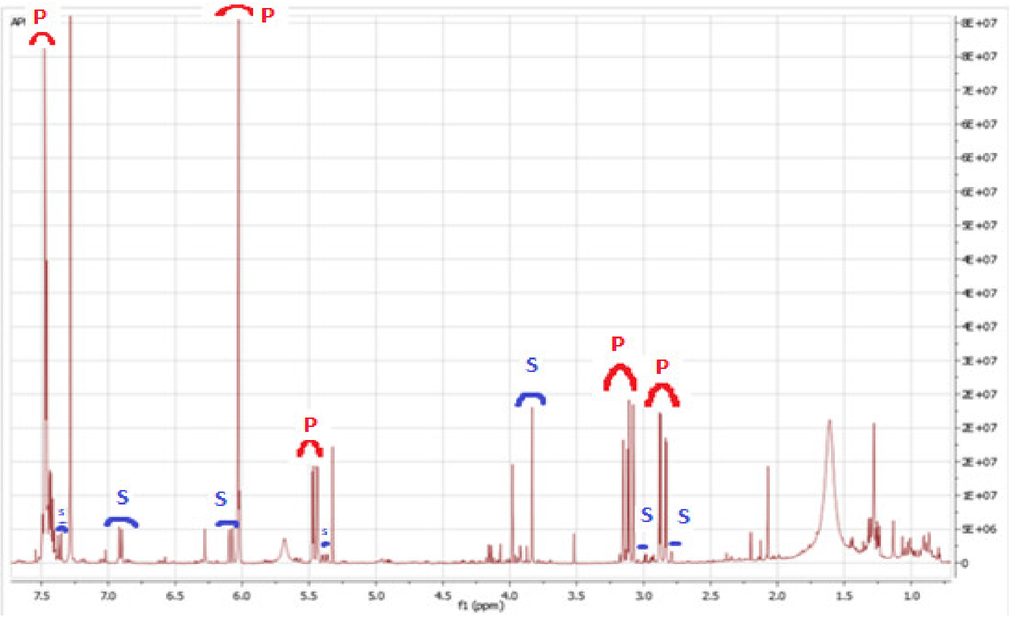

Figure S1: ^{1}H-NMR data of the mixture of P : pinocembrin and S : sakuranetin

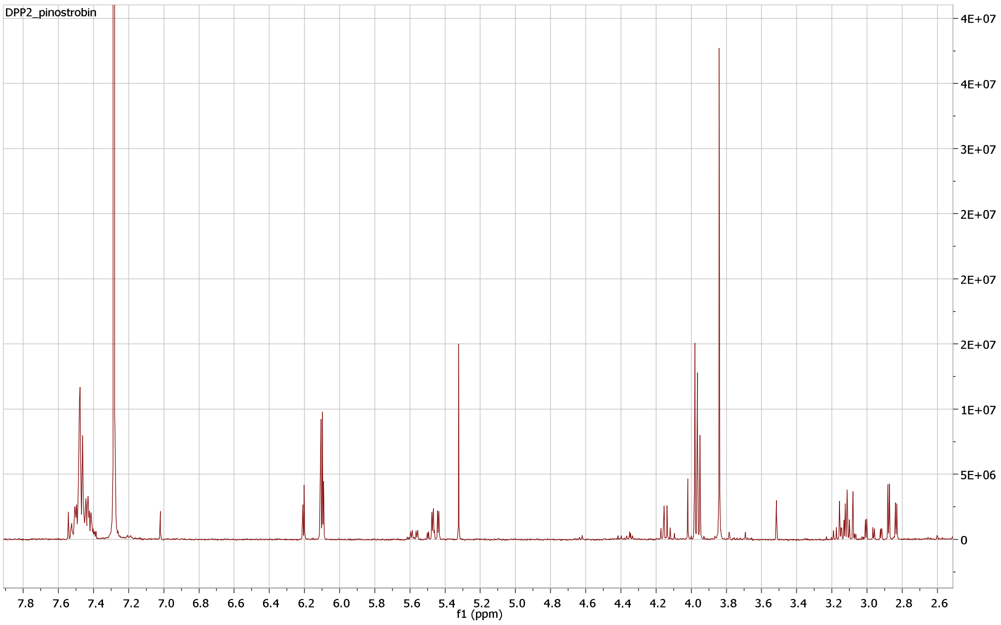

Figure S2: ^{1}H-NMR data of pinostrobin

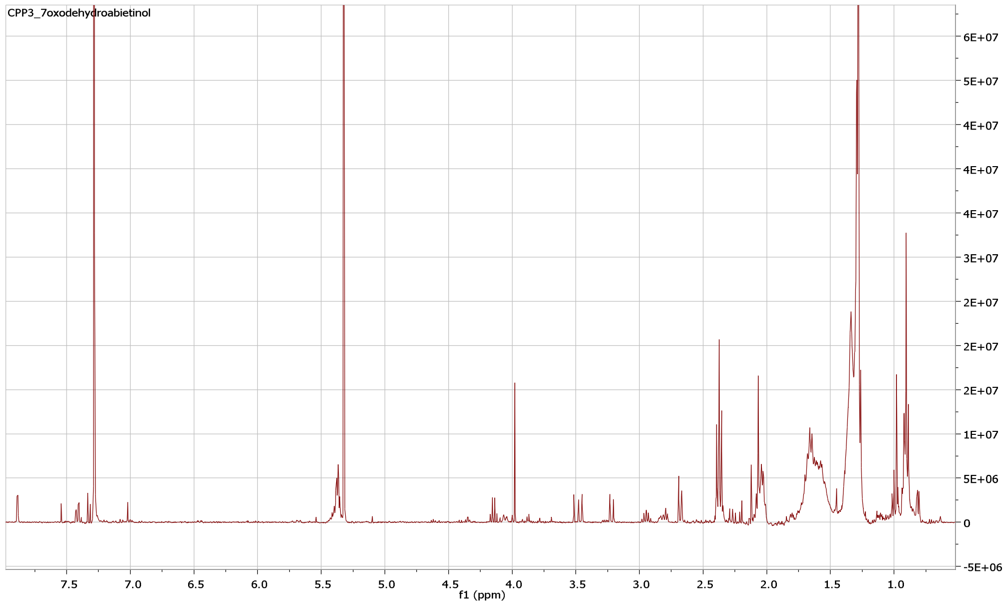

Figure S3: ^{1}H-NMR data of 7-oxo-dehydroabietinol

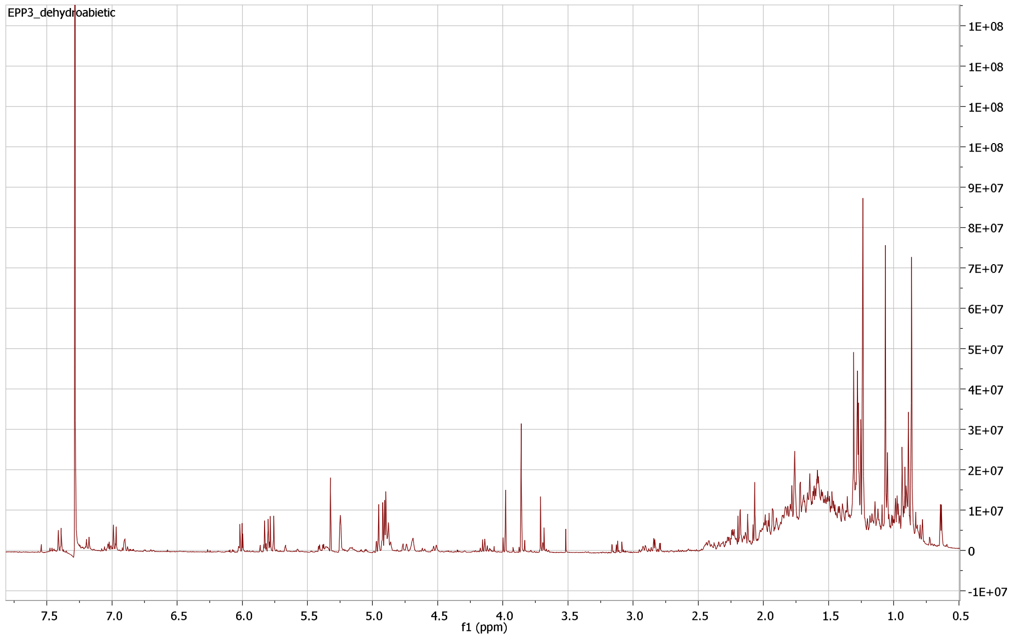

Figure S4: ^{1}H-NMR data of dehydroabietic acid

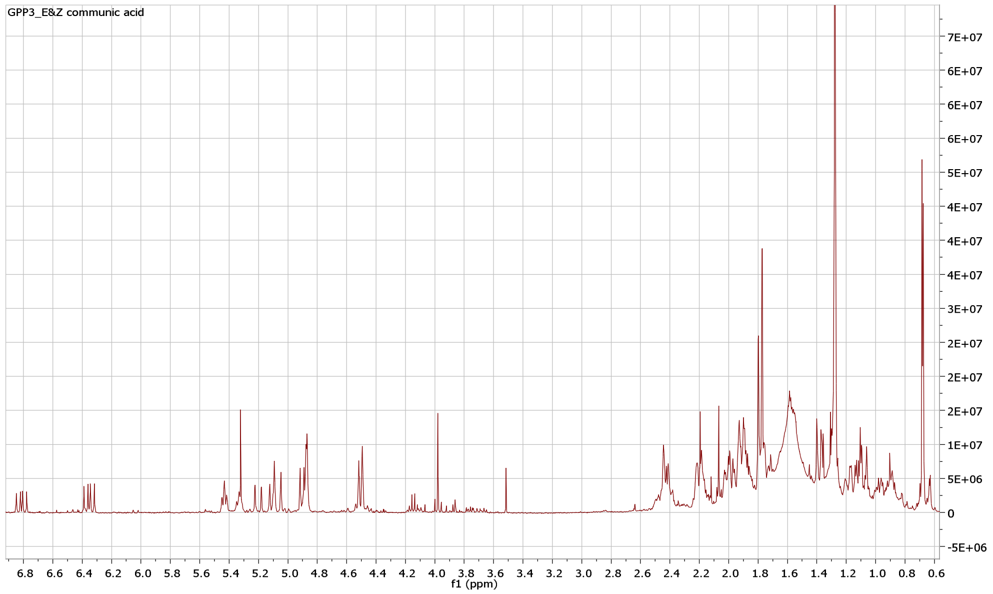

Figure S5: ^{1}H-NMR data of the mixture of *E*-communic acid and *Z*-communic acid

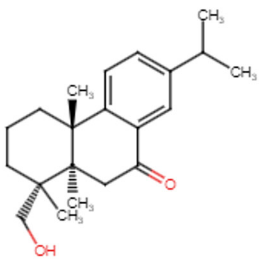

Formula S1: The structure of 7-oxo-dehydroabietinol
